# Exploring the comorbidity mechanisms of ITGB2 in rheumatoid arthritis and membranous nephropathy through integrated bioinformatics analysis

**DOI:** 10.1080/0886022X.2025.2536730

**Published:** 2025-07-23

**Authors:** Wenlong Cao, Yuqin Wang, Jing Xiong

**Affiliations:** a Department of Nephrology, Union Hospital, Tongji Medical College, Huazhong University of Science and Technology, Wuhan, China; b Department of Gynecologic Oncology, Women’s Hospital, School of Medicine, Zhejiang University, Hangzhou, China

**Keywords:** Rheumatoid arthritis, membranous nephropathy, macrophage polarization, B-cell activating factor, bioinformatics, machine learning

## Abstract

**Background:**

Patients with rheumatoid arthritis (RA) are more likely to comorbid renal diseases, with membranous nephropathy (MN) being the most common. This study aimed to explore the common pathogenesis between RA and MN using integrated bioinformatics analysis.

**Methods:**

Bulk and single-cell RNA sequencing datasets were obtained from the Gene Expression Omnibus and ImmPort databases. Differential expressed genes (DEGs) were identified and enrichment analysis was performed. Topology analysis and the random forest algorithm were applied to identify hub genes. The single-sample Gene Set Enrichment Analysis method was used to assess immune infiltration. Single-cell RNA sequencing analysis was employed to compare the transcript levels of key gene across different cell types. Pseudotime analysis was conducted using Monocle3, and cellular communication was analyzed with CellChat. The L1000FWD database was used to identify potential drugs, and molecular docking was performed.

**Results:**

66 common upregulated DEGs were identified, primarily associated with leukocyte migration and the chemokine signaling pathway. ITGB2 was finally identified as the shared pathogenic gene of both RA and MN. ITGB2 was predominantly expressed in macrophages, and its expression increased as M0 macrophages differentiated into M1 macrophages. BAFF signaling between macrophages with high ITGB2 expression and B cells/plasma cells was enhanced. Small molecules targeting ITGB2, including LY-294002 and CP466722, may serve as potential drugs for both RA and MN.

**Conclusion:**

As the pathogenic gene shared by both RA and MN, ITGB2 may play a role in M1 macrophage polarization and contribute to the maturation and differentiation of B cells through BAFF signaling.

## Introduction

As an immune-mediated glomerular disease, membranous nephropathy (MN) usually presents as nephrotic syndrome, characterized by massive proteinuria, hypoalbuminemia, edema, and hyperlipidemia. The pathological features of MN include thickening of the glomerular basement membrane and deposition of immune complex [1]. The global prevalence of MN is approximately 12 cases per 1,000,000 people, with 80% of cases being primary and 20% secondary [2]. In clinical practice, the prognosis of MN is often poor, with 20% of patients progressing to end-stage renal disease [3].

Rheumatoid arthritis (RA) is a chronic progressive multisystem inflammatory disease characterized by synovial inflammation and osteochondral damage [4,5]. The incidence of RA is approximately 0.5%–1%, with high recurrence rate, significant disability, and prolonged disease duration [6,7]. Although the etiology of RA remains unclear, both genetic and environmental factors play a significant role in its onset and progression [8]. Due to the chronic, inflammatory, and autoimmune nature of RA, patients may develop various extra-articular manifestations, including cardiovascular, respiratory, renal, neurological, gastrointestinal, and hematologic diseases [9–11]. Among these, renal involvement is relatively common and clinically significant, contributing to prolonged disease duration and increased mortality [12].

Several retrospective studies have shown that RA patients exhibit a variety of renal pathologies, including MN, renal amyloidosis, IgA nephropathy, and focal segmental glomerulosclerosis. Among these, MN is one of the most frequent renal pathologies in RA patients [13–16].

RA and MN are both autoimmune diseases. The occurrence of MN in RA patients has traditionally been attributed to the use of nonsteroidal anti-inflammatory drugs (NSAIDs) and disease-modifying antirheumatic drugs (DMARDs), such as gold salts, penicillamine, and bucillamine [14]. However, with advances in treatment strategies, numerous studies have reported that many RA patients with comorbid MN have no history of nephrotoxic drug use [17–19]. Additionally, a Mendelian randomization study based on large-scale Genome-Wide Association Studies data has shown that RA itself is a risk factor for MN [20]. Despite the above findings, the common pathogenesis between RA and MN remains unclear and warrants further investigation.

This study aims to explore the potential biomolecular mechanisms shared between RA and MN through integrated bioinformatics analysis. Our findings may offer new insights into the pathogenesis of both diseases.

## Materials and methods

### Data collection and preprocessing

Bulk RNA sequencing (bulk RNA-seq) datasets of RA (GSE7307, GSE206848, GSE77298) and MN (GSE108113) were obtained from the Gene Expression Omnibus (GEO) database (https://www.ncbi.nlm.nih.gov/geo/). Gene expression profiles were standardized and normalized using the ‘limma’ package [21]. The three bulk RNA-seq datasets of RA were merged and batch effects were removed using the ‘sva’ package [22].

Single-cell RNA sequencing (scRNA-seq) dataset of RA (SDY998) was retrieved from the ImmPort database (https://www.immport.org/), containing synovial tissue samples from 22 RA patients. The scRNA-seq dataset of MN (GSE131685) was obtained from GEO, including kidney tissue samples from 3 MN patients. Quality control and downstream analysis of scRNA-seq data were performed using the ‘Seurat’ package [23]. Cells of low quality were excluded based on the following criteria: 1) nFeature_RNA ≤ 200, 2) nFeature_RNA ≥ 7500, 3) percent.mt ≥ 30%, 4) percent.HB ≥ 5%, 5) nCount_RNA ≥ 100,000. nCount_RNA and nFeature_RNA reflect the sequencing depth of individual cell. Excessively high values of nCount_RNA and nFeature_RNA may indicate the presence of doublets while abnormally low values suggest empty droplets. percent.mt represents the proportion of mitochondrial genes, where elevated levels typically signify poor cell viability or even cell death. Similarly, percent.HB measures the proportion of erythrocyte-specific genes, which helps identify residual red blood cell contamination [24].

### Differentially expressed genes (DEGs) identification

The DEGs were identified using the ‘limma’ package [21] (*p* < 0.05 and |logFC|>1) and visualized using the ‘ggrepel’ package. A Venn diagram was employed to identify upregulated DEGs shared between MN and RA. The shared upregulated DEGs were visualized using the ‘pheatmap’ package.

### PPI network construction and enrichment analysis

The upregulated DEGs shared between RA and MN were used to explore their interactions using the Search Tool for the Retrieval of Interacting Genes/Proteins (STRING) online database (https://string-db.org/), with a median confidence score cutoff of 0.4. The protein-protein interaction (PPI) network was visualized and analyzed using Cytoscape (version 3.9.0) [25]. Gene Ontology (GO) and Kyoto Encyclopedia of Genes and Genomes (KEGG) analyses were performed using the ‘clusterProfiler’ package [26]. Four algorithms, including Maximal Clique centrality (MCC), Edge percolated component (EPC), Degree and Maximum Neighborhood Component (MNC), were used to screen the hub genes in the PPI network. The Intersection of the top 10 genes from all four algorithms was considered as hub genes.

### Feature selection with random Forest model

A random forest model was constructed using the ‘randomForest’ package and the importance score of each hub gene was calculated.

### Immune infiltration and correlation analysis

The single-sample Gene Set Enrichment Analysis (ssGSEA) was performed using 29 immune gene lists, which included genes related to various immune cell types, functions, pathways, and checkpoints. The ssGSEA algorithm was implemented *via* the ‘GSVA’ package to evaluate the immune characteristics of each sample in both MN and RA datasets [27]. The results were visualized as box plots using the ‘ggpubr’ package. Correlation analysis between ITGB2 expression and immune cell infiltration was also conducted.

### scRNA-seq analysis

Data normalization was performed using the ‘NormalizeData’ function followed by standardization using the ‘ScaleData’ function. The top 2000 highly variable genes were selected for principal component analysis (PCA). The ‘Harmony’ function (max.iter.harmony = 10, lambda = 1, theta = 2, sigma = 0.1) was applied to remove batch effects across samples [28]. The Uniform Manifold Approximation and Projection (UMAP) algorithm was used for dimensionality reduction and cluster identification at a resolution of 0.5. Cells were annotated based on markers from the CellMarker database [29] and previous literature (Supplementary Table 1). The ‘PercentageFeatureSet’ function was used to define ITGB2(+) and ITGB2(−) macrophages based on the percentage of ITGB2 expression (>0 and =0, respectively). Pseudotime analysis was conducted using the ‘Monocle3’ package to infer developmental trajectories and order cells based on transcriptional changes [30]. To explore the interactions and signaling pathways between ITGB2(+)/(−) macrophages and other cell types, the ‘CellChat’ package was employed [31].

### Candidate drugs identification and molecular docking

The L1000FWD database (https://maayanlab.cloud/l1000fwd/), a tool for drug discovery, was used to predict small molecule drugs for RA and MN. Compounds with an opposite correlation to the hub genes were identified as candidate drugs. To assess the reliability of drug-target binding, molecular docking was performed between candidate drugs and ITGB2. The mol2 files of the candidate drugs were obtained from the PubChem database (https://pubchem.ncbi.nlm.nih.gov/), while the 3D structure of ITGB2 was retrieved from the PDB database (https://www.rcsb.org/). Water molecules and heterogeneous molecules were removed using PyMol, and Chem3D was used to convert the 2D structures of small molecule drugs into 3D structures and optimize them for minimum free energy. AutoDock Tools 1.5.7 was employed for the hydrogenation and charge determination. Molecular docking was conducted using AutoDock Vina 1.1.2, and binding energies were calculated [32]. A lower binding energy indicates a more stable conformation between the ligand and receptor. A binding energy below −5 kcal/mol suggests fine binding activity, while a binding energy below −7 kcal/mol suggests strong binding activity. PyMol was used to visualize the docking results.

### Statistical analysis

Statistical analysis was performed using R software (version 4.4.0), with *p* < 0.05 considered statistically significant. A Wilcoxon test was used to compare ssGSEA results between diseased and normal samples. Additionally, Spearman’s correlation analysis was conducted to evaluate the relationship between biomarkers and ssGSEA results.

**Figure 1.**
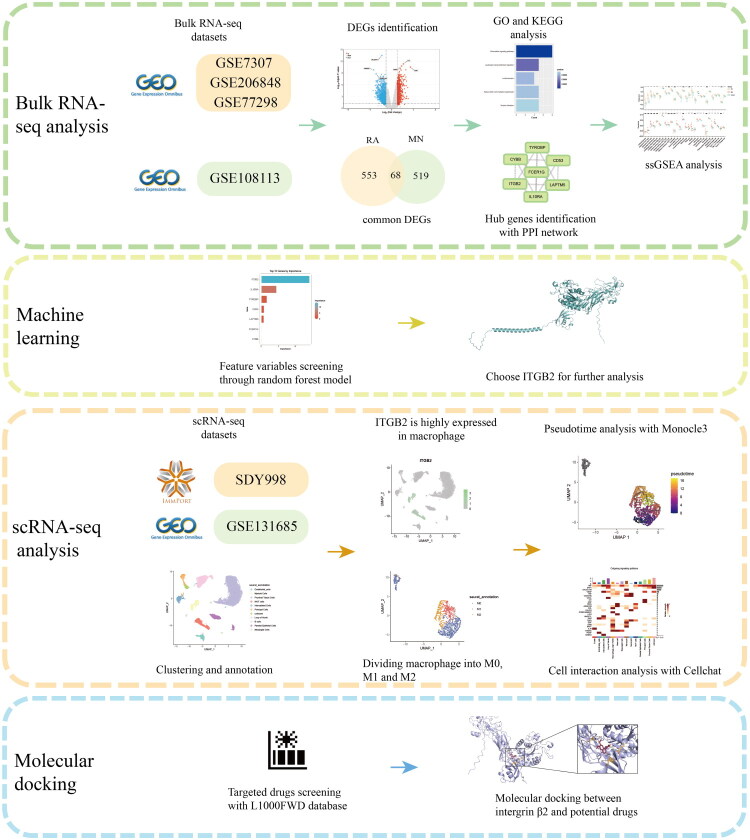
Flowchart of this study.

## Results

### Identification of common DEGs

**Figure 2.**
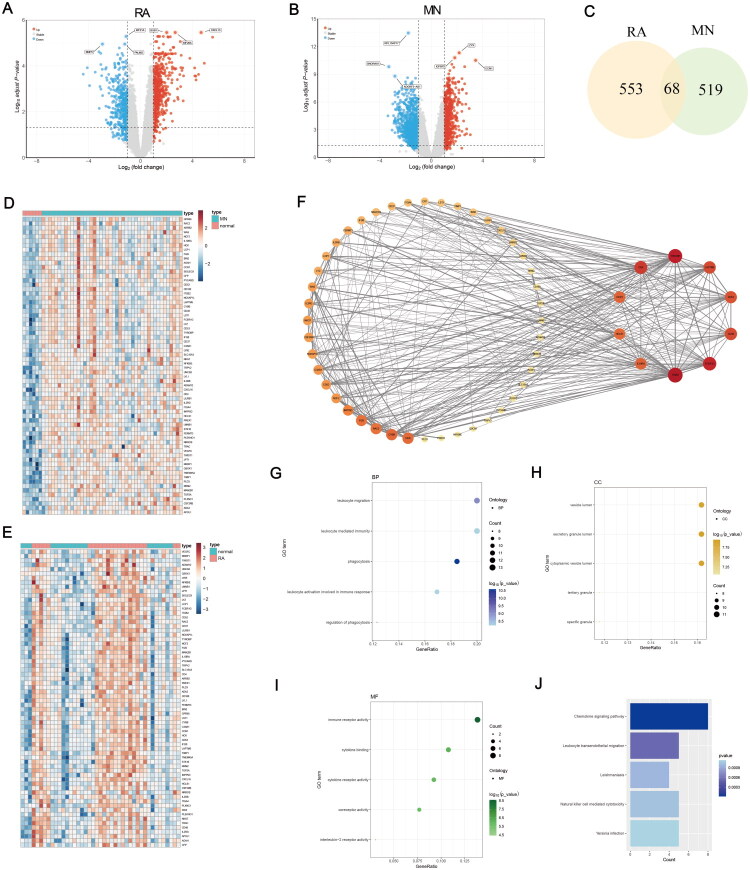
Identification of differentially expressed genes (DEGs) and functional enrichment analysis. (A and B) Volcano plots of DEGs in rheumatoid- arthritis (RA) and membranous nephropathy (MN) datasets. (C) Venn diagram to screen common DEGs in RA and MN. (D and E) Heatmaps of 68 common upregulated DEGs in RA and MN. (F) protein-protein interaction (PPI) network of 68 common upregulated DEGs. (G–I) Results of GO enrichment analysis results. (J) Results of KEGG pathway enrichment analysis.

The flowchart of this study was presented in Figure 1. After standardization and normalization, batch effects were evaluated and removed (Supplementary Figure 1A,B). Then, differential analysis was performed on the bulk RNA-seq datasets. A total of 1013 differential genes (587 upregulated and 426 downregulated) were identified in the merged RA dataset (Figure 2A). In the MN dataset (GSE108113), 1,538 differential genes (621 upregulated and 917 downregulated) were identified (Figure 2B). A total of 68 common upregulated DEGs were identified in both RA and MN (Figure 2C). The expression levels of these 68 common DEGs in each sample are displayed in heatmaps (Figure 2D,E).

### GO and KEGG analysis

The 68 common DEGs were selected for GO and KEGG pathway enrichment analysis to explore their biological functions. The GO analysis revealed that the most significant processes and components included leukocyte migration (biological process) (Figure 2G), vesicles (cellular component) (Figure 2H), and immunoreceptor activity (molecular function) (Figure 2I). KEGG pathway analysis indicated that these 68 common DEGs were primarily involved in the chemokine signaling pathway (Figure 2J).

### Hub gene identification

**Figure 3.**
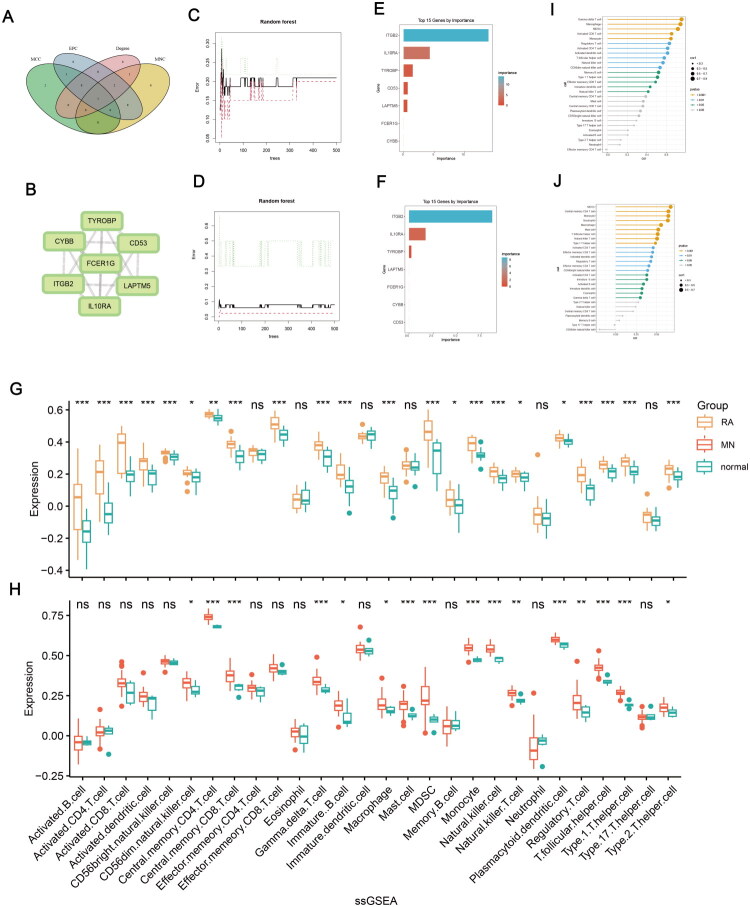
Screening of the key gene and immune infiltration analysis. (A) Venn diagram to intersect the results from four topology algorithms (MCC, EPC, degree, MNC). (B) PPI network of 7 common hub genes in RA and MN. (C and D) Correlation between the total number of trees and the error rate in RA and MN datasets based on random Forest algorithm. (E and F) Relative importance scores of 7 hub genes in RA and MN. (G and H) Comparison of immune cell infiltration between diseased and normal samples in RA and MN. (I and J) Correlation analysis between the key gene (ITGB2) expression and immune cell infiltration. ns: no significance, *p < 0.05, **p < 0.01, ***p < 0.001.

The 68 common DEGs were imported into STRING online database to construct the PPI network, which was analyzed and visualized with Cytoscape (Figure 2F). Seven hub genes were identified by intersecting the results from four algorithms including MCC, EPC, Degree and MNC (Figure 3A). The seven hub genes identified were ITGB2, FCER1G, TYROBP LAPTM5, CYBB, IL10RA, and CD53 (Figure 3B).

### Key gene screening with machine learning

The random forest model results for both RA and MN datasets revealed that ITGB2 ranked first in the importance ranking of all hub genes (Figure 3C–F). We further compared the ITGB2 expression level across individual dataset. In GSE7307, GSE77298 and GSE108113, ITGB2 expression was significantly elevated in disease groups versus controls. However, no statistical significance was observed in GSE206848, which might be attributed to its small sample size (Supplementary Figure 2A–D). These suggest that ITGB2 may be a key gene involved in the pathogenesis of both RA and MN, and it was therefore selected for further analysis.

### Immune infiltration analysis

It was demonstrated in the results of immune infiltration analysis that both the RA and MN samples showed higher expression of various immune cells, including CD56 bright natural killer cell, central memory CD4 T cell, central memory CD8 T cell, gamma delta T cell, immature B cell, macrophage, myeloid-derived suppressor cell (MDSC), monocyte, natural killer cell, natural killer T cell, plasmacytoid dendritic cell, regulatory T cell, T follicular helper cell, type 1 T helper cell, type 2 T helper cell (Figure 3G,H). ITGB2 expression was significantly correlated with the infiltration of MDSC, monocyte and macrophage in both RA and MN (Figure 3I,J).

### RA scRNA-seq analysis

**Figure 4.**
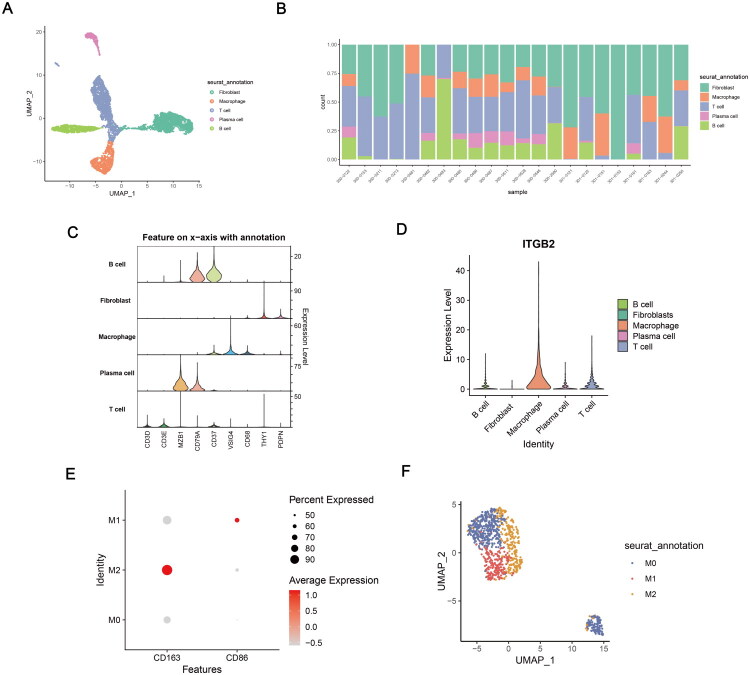
Overview of the scRNA-seq data of RA. (A) The UMAP plot classified by cell type. (B)Proportional representation of each cell type in RA samples. (C) Expression of gene markers in different cells. (D) ITGB2 expression in different cells. (E) Expression of markers associated with macrophage polarization. (F) The UMAP plot of macrophages classified by cell type.

After filtering and quality control, 7071 cells from the RA scRNA-seq dataset were analyzed (Supplementary Figure 3A). Then, batch effects were evaluated and removed (Supplementary Figure 1C,D). Fibroblasts, macrophages, T cells, plasma cells, and B cells were identified (Figure 4A–C). ITGB2 showed the highest expression in macrophages (Figure 4D). Macrophages were further classified into M0, M1, and M2 subtypes (Figure 4E,F).

### MN scRNA-seq analysis

**Figure 5.**
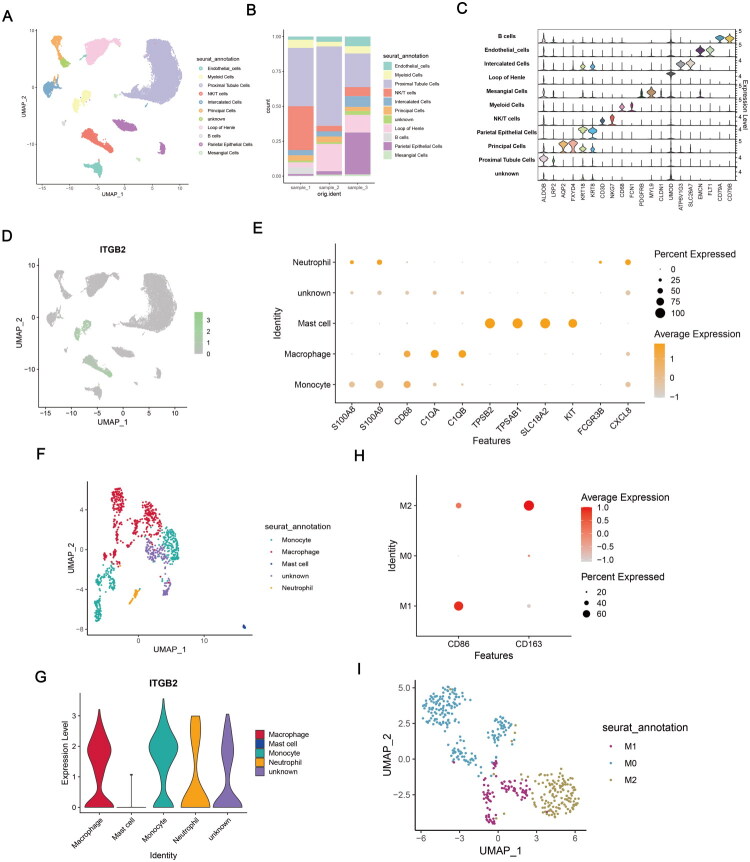
Overview of the scRNA-seq data of MN. (A) The UMAP plot classified by cell type. (B) Proportional representation of each cell type in MN samples. (C) Expression of gene markers in different cells. (D) Expression of ITGB2 in different cells. (E) Expression of gene markers in different myeloid cells. (F) The UMAP plot of myeloid cells classified by cell type. (G) ITGB2 expression in different myeloid cells. (H) Expression of markers associated with macrophage polarization. (I) The UMAP plot of macrophages classified by cell type.

After filtering and quality control, 24,060 cells from the MN scRNA-seq dataset were analyzed (Supplementary Figure 3B–D). Then, batch effects were evaluated and removed (Supplementary Figure 1E,F). Endothelial cells, myeloid cells, proximal tubular cells, NK/T cells, intercalated cells, principal cells, loop of henle, B cells, and mesangial cells were identified (Figure 5A–C). ITGB2 exhibited the highest expression in myeloid cells and NK/T cells (Figure 5D). Myeloid cells were further divided into monocytes, macrophages, mast cells, and neutrophils (Figure 5E,F). ITGB2 was highly expressed in monocytes, macrophages and neutrophils (Figure 5G). Macrophages were further classified as M0, M1, or M2 subtypes (Figure 5H,I).

### Pseudotime analysis

**Figure 6.**
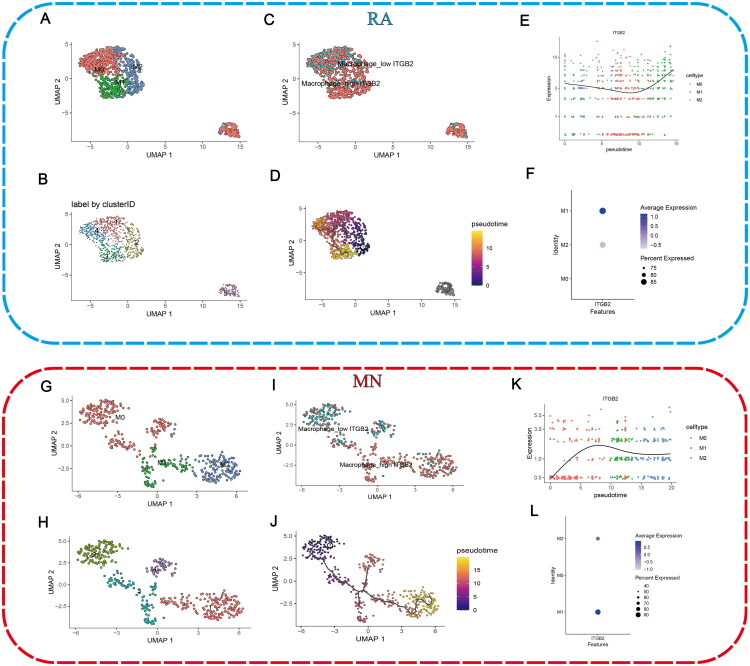
Pseudotime analysis of macrophage differentiation in RA and MN. (A–D, G–J) Pseudotime trajectory of macrophages. (E and K) Change of ITGB2 expression with pseudotime trajectory in different types of macrophages. (F and L) Expression of ITGB2 in different types of macrophages.

Macrophage differentiation trajectories were simulated using the Monocle3 algorithm (Figure 6A–D,G–J). In RA, ITGB2 expression increased as M0 macrophages differentiated into M2 macrophages along the pseudotime trajectory. ITGB2 expression increased more significantly as M0 macrophages transformed into M1 macrophages (Figure 6E). In MN, ITGB2 expression increased as M0 macrophages differentiated into M1 macrophages. While M1 macrophages transformed into M2 macrophages, ITGB2 expression decreased (Figure 6K). In both RA and MN, ITGB2 expression was significantly higher in M1 macrophages compared to M2 and M0 macrophages (Figure 6F,L).

### Cellular communication analysis

**Figure 7.**
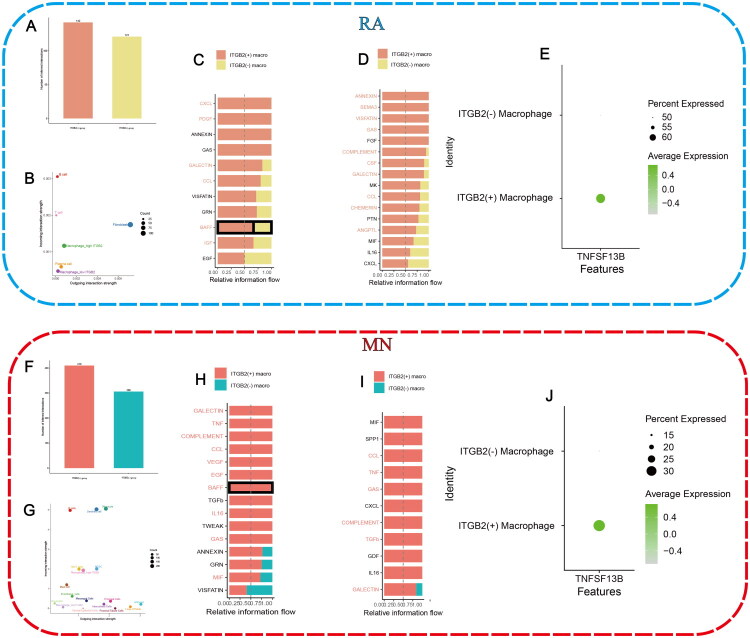
Cellular communication analysis in RA and MN. (A and F) Comparison of overall intensity of cellular communication between ITGTB2(+) group and ITGTB2(−) group. ITGB2(−) macrophages were not included in ITGB2(+) group while ITGB2(+) macrophages were not included in ITGB2(−) group. (B and G) Intensity of signals in each cell type. (C, D, H, I) Differences in cellular communication between the two groups when macrophages act as signal receivers or senders. (E and J) Expression of TNFSF13B in different types of macrophage.

ITGB2(+) macrophages demonstrated significantly higher signaling intensity than ITGB2(-) macrophages in both RA and MN (Figure 7A,B,F,G), acting as senders (GALECTIN, CCL, BAFF; Figure 7C,H) and receivers (CCL, GAS, COMPLEMENT, GALECTIN; Figure 7D,I). The intensity of signaling pathway was visualized as heatmaps (Supplementary Figure 4A–H). Among the above signaling pathways, the BAFF signaling pathway is crucial for the survival, proliferation, and differentiation of B cells. TNFSF13B, which encodes the B-cell activating factor (BAFF), was upregulated in ITGB2(+) macrophages in both RA and MN, enhancing interactions between macrophages and B cells (TNFSF13B-TNFRSF13C) or plasma cells (TNFSF13B-TNFRSF17) (Figure 7E,J).

### Molecular docking of drug candidates

**Figure 8.**
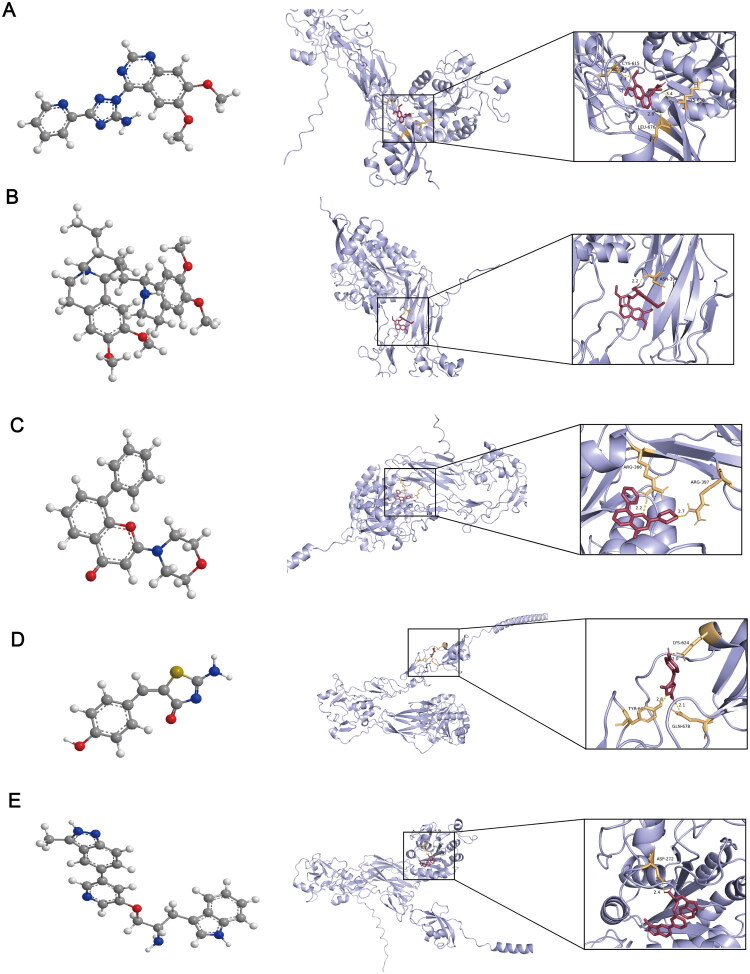
Molecular docking of candidate small molecule drugs with ITGB2. 3D structures and molecular docking of (A) CP466722, (B) emetine, (C) LY-294002, (D) mirin and (E) A-443644.

Five candidate drugs were screened from the L1000FWD database (Table 1): CP466722 (Figure 8A), emetine (Figure 8B), LY-294002 (Figure 8C), mirin (Figure 8D), and A-443644 (Figure 8E). These drugs were predicted to bind to ITGB2 through hydrogen bonding. The binding energies of these five drugs are shown in Table 2. Among them, LY-294002 (-7.8 kcal/mol) and CP466722 (−7.8 kcal/mol) demonstrated the lowest binding energies, suggesting the most stable binding interactions with ITGB2.

**Table 1.** Drugs of opposite relevance in the L1000FWD.

Rank	Compound	Similarity score	*p* value	*Q* value	Z-score
1	CP466722	−0.7143	4.20e-08	3.69e-05	1.64
2	emetine	−0.7143	2.92e-08	3.69e-05	1.80
3	LY-294002	−0.7143	3.49e-08	3.69e-05	1.82
4	mirin	−0.7143	3.30e-08	3.69e-05	1.74
5	A-443644	−0.7143	4.67e-08	3.69e-05	1.63

**Table 2.** Molecular docking of candidate drugs with ITGB2.

Compound	Receptor	Affinity(kcal/mol)	Hydrogen bond
CP466722	ITGB2	−6.7	CYS-615, LYS-658, LEU-676
Emetine	ITGB2	−6.3	ASN-394
LY-294002	ITGB2	−7.5	ARG-366, ARG-397
Mirin	ITGB2	−5.8	LYS-624, TYR-607, GLN-678
A-443644	ITGB2	−6.4	ASP-272

## Discussion

MN is an immune-relevant kidney disease with various target antigens identified, including phospholipase A2 receptor, thrombospondin type 1 domain-containing 7 A, and neural epidermal growth factor-like 1 protein [33,34]. RA is a multi-system autoimmune disease characterized by erosive arthritis. Extra-articular involvement in RA can affect several organs, including the heart, lung, and kidney. The development of extra-articular complications significantly contributes to poor prognosis in RA patients [4,5] while MN is one of the most common kidney diseases observed in RA patients. While DMARDs such as gold salts, penicillamine, and bucillamine can induce secondary MN, many RA patients who have MN do not have a history of nephrotoxic drug use [35]. Therefore, the common autoimmune-relevant pathogenesis between RA and MN remains unclear. In this study, we aimed to utilize bioinformatics analysis to explore the potential comorbidity mechanisms between RA and MN.

From the bulk RNA-seq data, 68 common upregulated DEGs in both MN and RA were identified. Enrichment analysis revealed that these DEGs were predominantly involved in leukocyte migration and the chemokine signaling pathway. Seven hub genes were screened from the PPI network, including ITGB2, FCER1G, TYROBP, LAPTM5, CYBB, IL10RA, and CD53. Based on the random forest model’s importance scores, ITGB2 was identified as a key gene potentially involved in the pathogenesis of both RA and MN. A previous study demonstrated elevated ITGB2 expression in glomerular region of MN patients, aligning with our scRNA-seq results [36].

Integrin β2 (ITGB2), also known as CD18, is primarily expressed on the surface of leukocytes, where it plays a crucial role in leukocyte adhesion and migration through interactions with integrin α. Immune infiltration analysis revealed that ITGB2 expression was highly correlated with the infiltration of MDSC, monocyte, and macrophage in both RA and MN. These findings suggest that ITGB2 plays a pivotal role in immune cell infiltration during these diseases. scRNA-seq analysis further confirmed that ITGB2 was highly expressed in macrophages in both RA and MN, aligning with the immune infiltration results. Primitive macrophages can differentiate into pro-inflammatory M1 macrophages or anti-inflammatory M2 macrophages in response to various environmental signals. Our results showed that ITGB2 was predominantly expressed in M1 macrophages in both RA and MN, with lower expression in M0 and M2 macrophages. To assess the dynamic changes in ITGB2 expression during macrophage differentiation, pseudotime analysis was performed. ITGB2 expression increased as M0 macrophages differentiated toward M1 macrophages, suggesting that ITGB2 may be involved in M1 macrophage polarization. Renal injury and fibrogenic effects of M1 macrophages have been reported [37,38]. In addition, in RA patients, cartilage damage can be caused by M1 macrophages due to the inflammatory cytokines [39].

Cellular communication analysis revealed that ITGB2(+) macrophages exhibited enhanced interactions with other cell types compared to ITGB2(−) macrophages. Notably, the interaction between ITGB2(+) macrophages and B cells/plasma cells *via* BAFF signaling was significantly upregulated. BAFF belongs to the tumor necrosis factor super family and plays a critical role in the maturation and survival of B cell and plasma cell. BAFF exerts its effects by binding to three receptors: BAFFR (B-cell activating factor receptor), TACI (Tumor necrosis factor receptor superfamily member 13B), and BCMA (B cell maturation antigen). BAFFR is involved in the maturation of naive B cells [40], while TACI promotes the differentiation of activated B cells toward plasmablasts and antibody class switching [41]. BCMA is expressed on plasma cells and is essential for their survival [42]. B cells function as promoting the development of T cells by presenting the antigens and differentiating into plasma cells to secrete antibodies [43,44]. Overactivation of B cells has been identified as a key pathogenic factor for RA and MN. Rituximab, an anti-CD20 monoclonal antibody targeting B cells, is recommended as a first-line treatment for MN by the Kidney Disease: Improving Global Outcomes guidelines [45], and as a second-line therapy for RA by the American College of Rheumatology and the European League Against Rheumatism [46,47]. BAFF inhibitors such as belimumab and telitacicept have been applied in small prospective clinical researches to evaluate their therapeutic effect in RA [48] and MN [49,50].

Additionally, small molecules inversely correlated with the hub genes were screened from the L1000FWD database, including CP466722, emetine, LY-29400, mirin, and A-443644. Results of molecular docking indicated that CP466722 and LY-294002 exhibited the most stable binding to ITGB2. CP466722, an ATM kinase inhibitor, may mitigate inflammatory kidney injury by downregulating the NF-κB signaling pathway [51]. LY-294002, which inhibits the PI3K/AKT/mTOR pathway, has been shown to reduce podocytes apoptosis in MN [52]. These findings suggest that these small molecules may represent potential therapeutic options for RA and MN through targeting ITGB2.

It is the first bioinformatics research into the comorbidity mechanisms between RA and MN. However, there are some limitations. Lack of clinical metadata in the analyzed datasets limited our ability to study the relationship between the hub gene and disease duration as well as therapy effects. In addition, the comorbidity mechanisms between RA and MN and therapeutic effects of the identified small molecule drugs require further validation through experimental research.

## Conclusion

ITGB2 appears to play a central role in the pathogenesis of both RA and MN by promoting M1 macrophages polarization and enhancing BAFF signaling, which drives the overactivation of B cells. Small molecules targeting ITGB2, such as LY-294002 and CP466722, are potential drugs for the treatment of both RA and MN. Our findings provide new insights into potential treatment strategies for these diseases.

## Supplementary Material

Fig S1.tif

Fig S4.tif

Fig S3.tif

Fig S2.tif

## Data Availability

All data generated or analyzed during this study are included in this published article.
