# Supplementary figures and images for: Exploring the comorbidity mechanisms of ITGB2 in rheumatoid arthritis and membranous nephropathy through integrated bioinformatics analysis
Source: Ren Fail. 2025 Jul 23;47(1):2536730. doi: 10.1080/0886022X.2025.2536730 (PMC12288179; doi:10.1080/0886022X.2025.2536730)

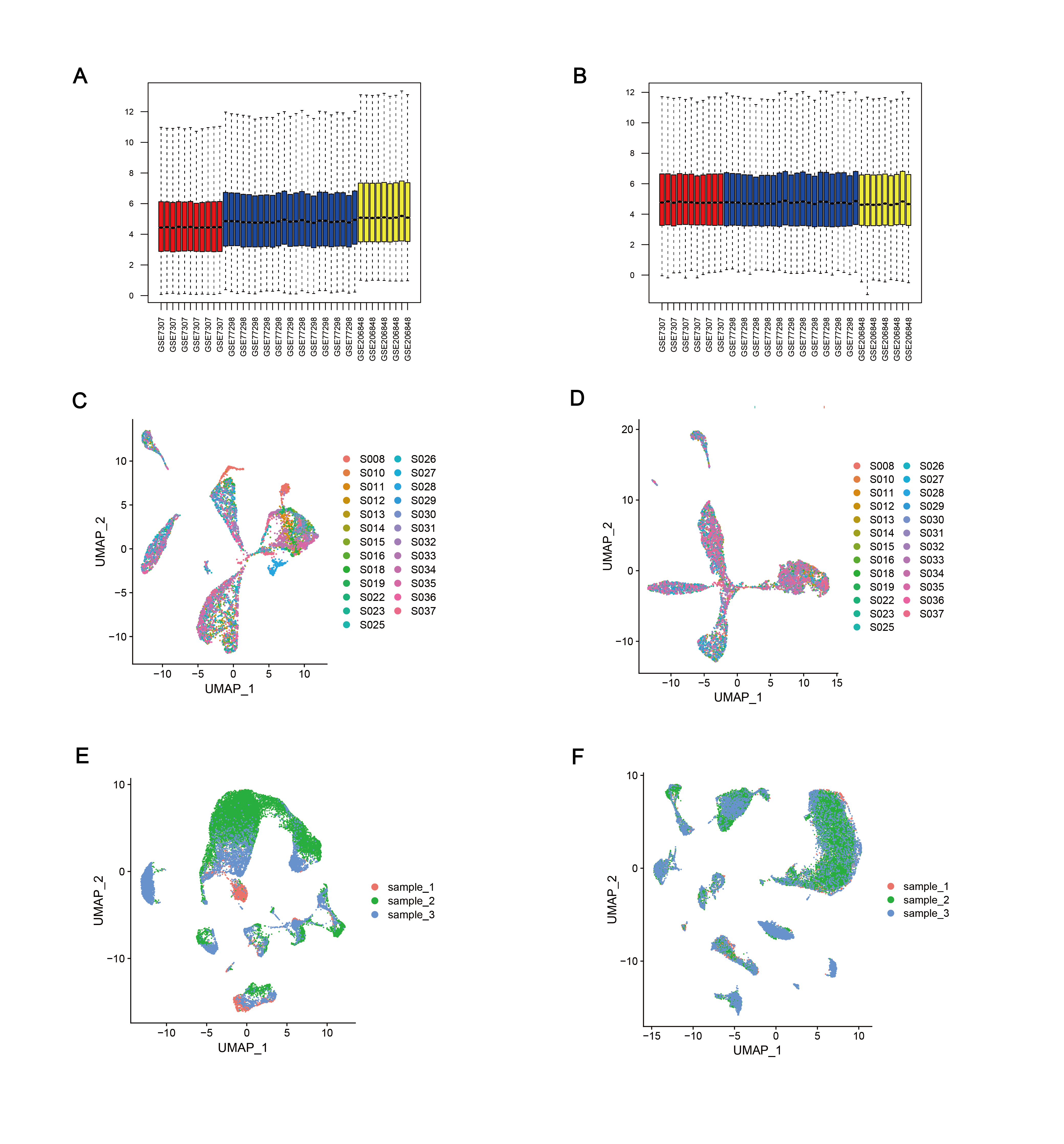

Supplement: Fig S1.tif [file IRNF_A_2536730_SM7606.tif]

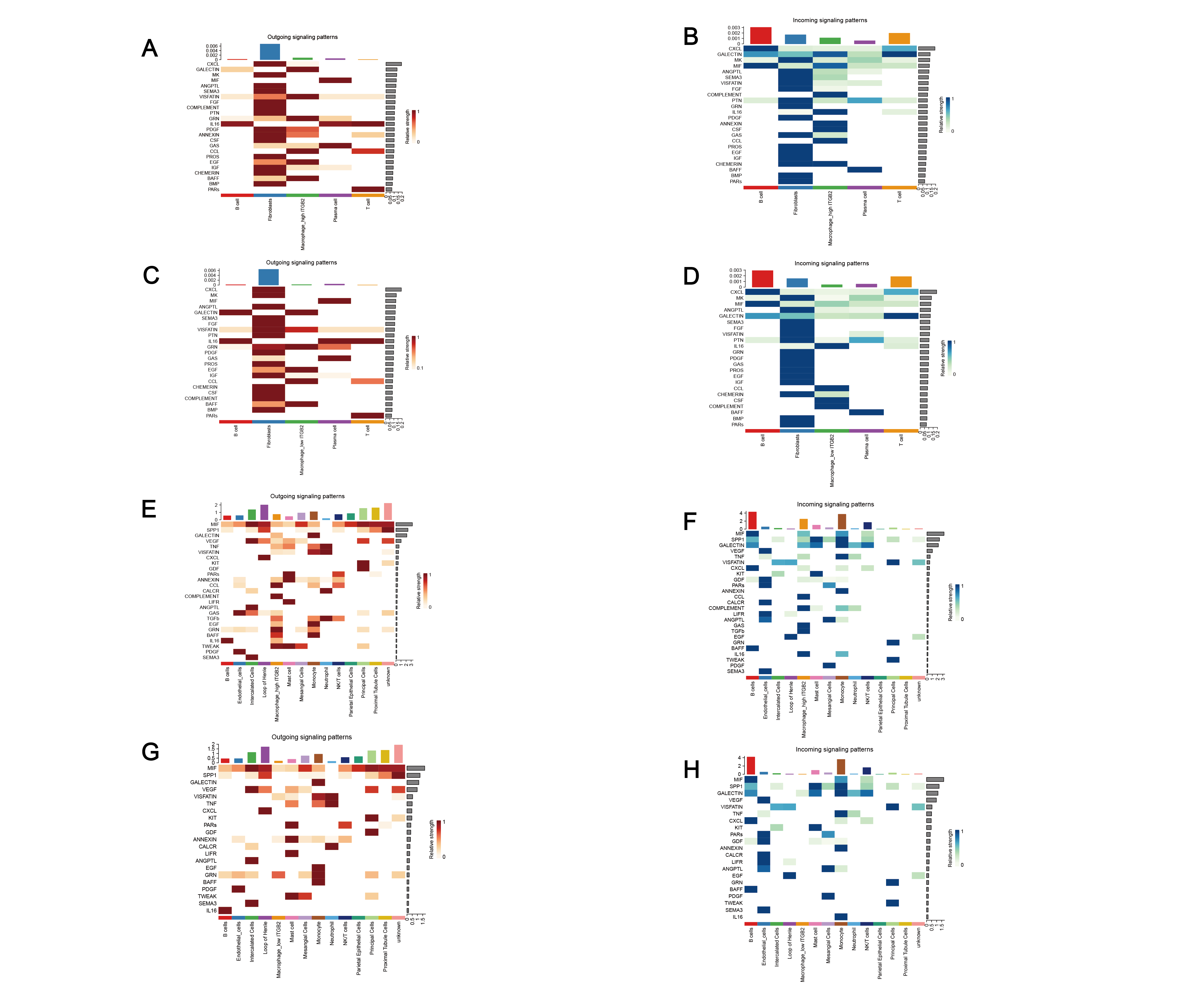

Supplement: Fig S4.tif [file IRNF_A_2536730_SM7605.tif]

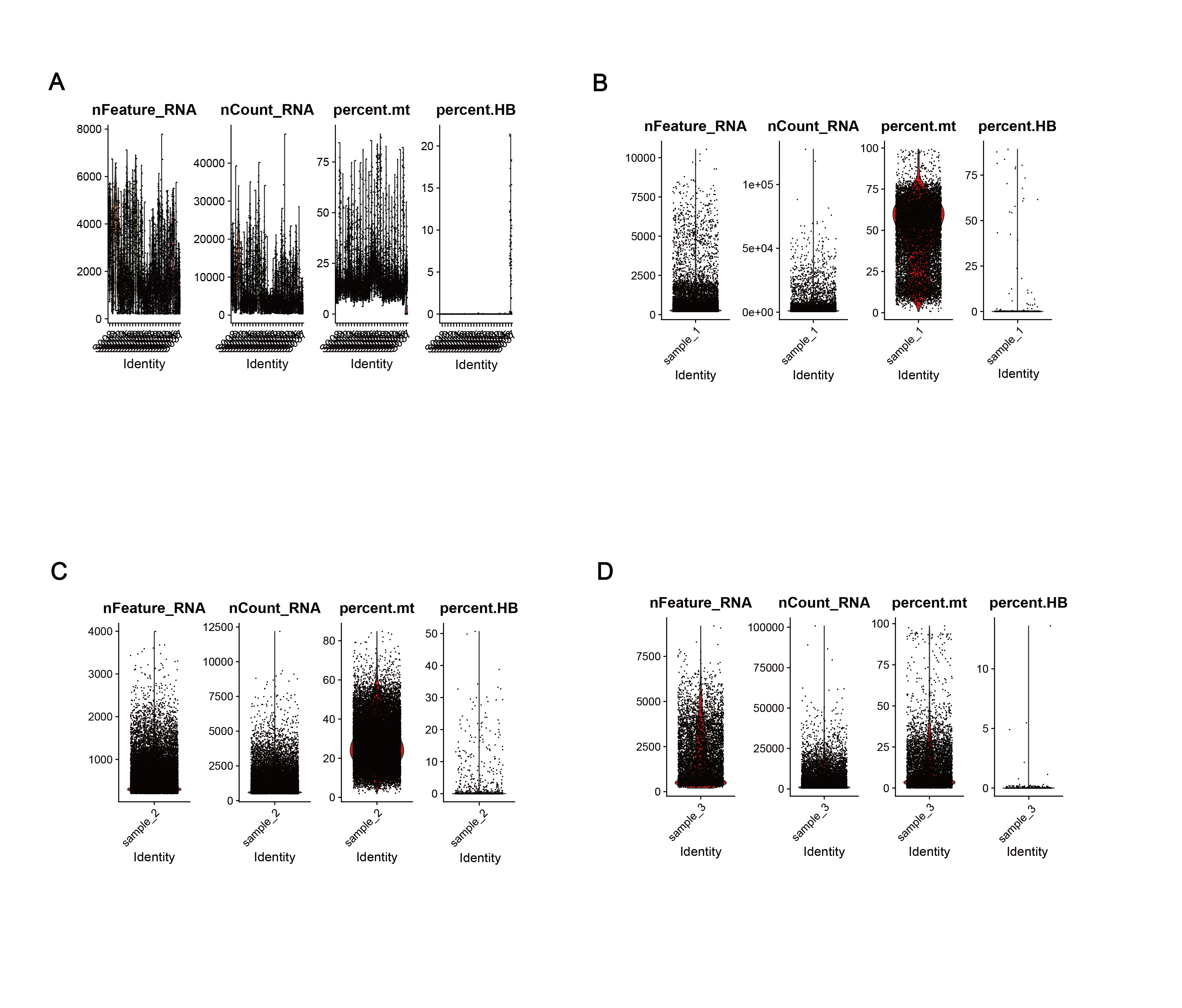

Supplement: Fig S3.tif [file IRNF_A_2536730_SM7604.tif]

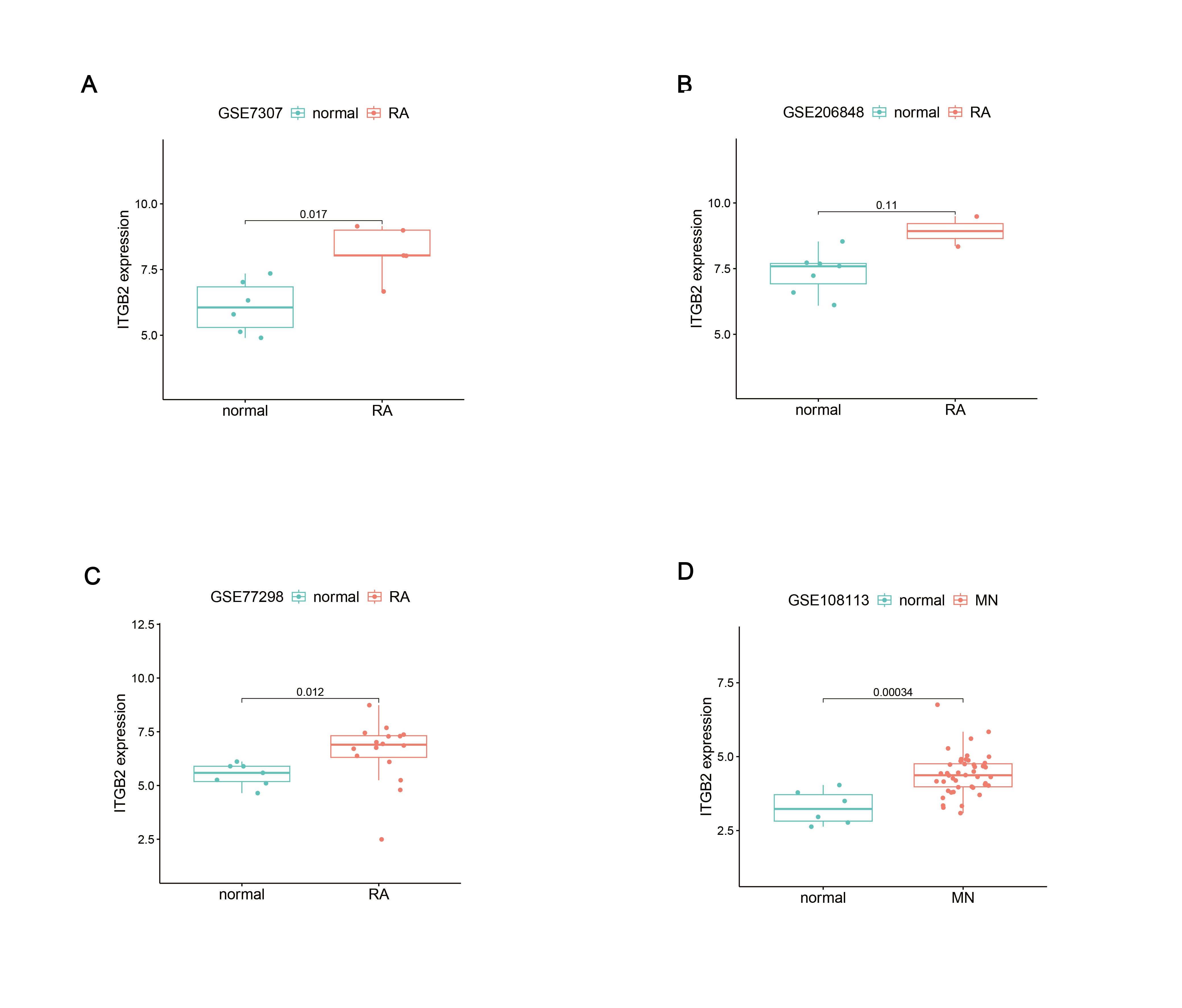

Supplement: Fig S2.tif [file IRNF_A_2536730_SM7602.tif]
